# Modeling the effects of vaccination, nucleic acid testing, and face mask wearing interventions against COVID-19 in large sports events

**DOI:** 10.3389/fpubh.2022.1009152

**Published:** 2022-11-09

**Authors:** Zeting Liu, Huixuan Zhou, Ningxin Ding, Jihua Jia, Xinhua Su, Hong Ren, Xiao Hou, Wei Zhang, Chenzhe Liu

**Affiliations:** ^1^Department of Mathematic Science, School of Sport Engineering, Beijing Sport University, Beijing, China; ^2^Department of Physical Fitness and Health, School of Sport Science, Beijing Sport University, Beijing, China; ^3^Key Laboratory of Sports and Physical Health, Ministry of Education, Beijing Sport University, Beijing, China; ^4^School of Government, Wellington School of Business and Government, Victoria University of Wellington, Wellington, New Zealand; ^5^Department of Chemical Drug Control, China National Institute for Food and Drug Control, Beijing, China

**Keywords:** COVID-19 vaccine, COVID-19 nucleic acid testing, mathematical model, SEIR model, Wells–Riley model

## Abstract

The transmission of SARS-CoV-2 leads to devastating COVID-19 infections around the world, which has affected both human health and the development of industries dependent on social gatherings. Sports events are one of the subgroups facing great challenges. The uncertainty of COVID-19 transmission in large-scale sports events is a great barrier to decision-making with regard to reopening auditoriums. Policymakers and health experts are trying to figure out better policies to balance audience experiences and COVID-19 infection control. In this study, we employed the generalized SEIR model in conjunction with the Wells–Riley model to estimate the effects of vaccination, nucleic acid testing, and face mask wearing on audience infection control during the 2021 Chinese Football Association Super League from 20 April to 5 August. The generalized SEIR modeling showed that if the general population were vaccinated by inactive vaccines at an efficiency of 0.78, the total number of infectious people during this time period would decrease from 43,455 to 6,417. We assumed that the general population had the same odds ratio of entering the sports stadiums and becoming the audience. Their infection probabilities in the stadium were further estimated by the Wells–Riley model. The results showed that if all of the 30,000 seats in the stadium were filled by the audience, 371 audience members would have become infected during the 116 football games in the 2021 season. The independent use of vaccination and nucleic acid testing would have decreased this number to 79 and 118, respectively. The combined use of nucleic acid testing and vaccination or face mask wearing would have decreased this number to 14 and 34, respectively. The combined use of all three strategies could have further decreased this number to 0. According to the modeling results, policymakers can consider the combined use of vaccination, nucleic acid testing, and face mask wearing to protect audiences from infection when holding sports events, which could create a balance between audience experiences and COVID-19 infection control.

## Introduction

The outbreak of COVID-19 has affected 222 countries and regions worldwide and has led to 102 million cases and 2.2 million deaths (1). Its pathogen, SARS-CoV-2, has a higher transmissibility than H1N1 and SARS-CoV (2). The pathogen mainly transmits *via* respiratory droplets and contact. Social gathering and migration are the primary reasons for the spread of SARS-CoV-2. To prevent the transmission of COVID-19, the governments of some countries have implemented a series of emergency measures, such as travel restrictions and the cessation of social gathering events, which successfully contain the transmission of COVID-19 while inevitably limiting the development of some of the industries relying on social gathering and casting a pall over participants (3).

Sports events are one of the subgroups facing great challenges in the post-pandemic era. Some games were played in an empty stadium, while other stadiums opened a small part of their auditorium to maintain safe social distancing. Home-and-away games were prohibited. These measures have had some negative effects on the development of sports economics and have impaired audience experiences. For example, the Chinese Football Association Super League (hereinafter referred to as CSL), one of the most prosperous professional sports events in China, attracted a total of 5.6 million audience members (25,000–30,000 audience members for each game) in the 2019 game season (4). However, the CSL has been played in an empty stadium since the outbreak of COVID-19. Without match day revenue, 16 professional football teams announced their intention to disband, and Jiangsu Suning F.C., the champion team of 2020 CSL, announced its closing down due to financial crisis in 2021. The experience of millions of fans has been impaired at the same time. Therefore, it is an urgent task for policymakers to establish some policies other than social distancing to control COVID-19 infection and reopen sports events to the audience.

Vaccination against COVID-19 and nucleic acid testing provide the potential to resume attending sports events. The Chinese residents aged 3 years or more have been encouraged to receive a two-dose inoculation by inactivated vaccines since September 2020, based on a high vaccine supply and the safety reported by a series of clinical trials (5–8). The CSL tried to reopen a couple of games to the audience in 2021. The allowed admission was decreased from 30,000 in the pre-pandemic era to 2,000. The audiences were required to show a negative record of nucleic acid testing within 7 days prior to the games (7) and to wear face masks when watching them. Meanwhile, to avoid the transmission of COVID-19 in the transportation of audiences and football teams, games were held in two cities instead of being home-and-away games. As a result, the income associated with the CSL and the audience experiences were not substantially enhanced toward the pre-pandemic levels. The uncertainty of COVID-19 transmission among audiences is a great barrier for policymakers to make a decision with regard to resuming sports events with large crowds.

Dynamic models of infection can be utilized to predict the effects of preventive strategies against transmission (9–12). Compared to the Susceptible, Exposed, Infectious (SIR) model and the classic Susceptible, Exposed, Infectious, Recovered (SEIR) model, the generalized SEIR model (13) is more appropriate to reflect the transmission dynamics of COVID-19 (14), which accounts for some important characteristics of the disease, such as the latency period, death, quarantine for confirmed cases, and the protection rate of susceptible populations. Some efforts have been devoted to predict the effectiveness of various preventive strategies against COVID-19 transmission in the general population by the generalized SEIR model (15–20). By adjusting the protection rate of the SEIR model, the impact of vaccination (15–20) and non-pharmaceutical interventions (NPIs) (15, 16, 18, 20) have been simulated. The results of some studies suggest that the effectiveness of vaccination depends on the efficacy of the vaccines used and the coverage of vaccination (15, 17–19); if the population cannot be fully covered by effective vaccination, combining vaccination and NPIs is an ideal measure to reduce the number of confirmed cases and deaths of COVID-19 (15, 16, 20). These studies have indicated that the generalized SEIR model is useful for simulating COVID-19 dynamics in the general population during a long period of time, and the herd immunity effect of vaccination can be predicted. However, to predict the transmission probability in audiences during a sports event, we need to further simulate a scenario in which susceptible people could be infected through air droplets in a confined space.

The Wells–Riley model is based on the concept of “quantum of infection,” which is defined as the dose of pathogens required to infect a susceptible person when he or she inhales in a ventilated room during a time of exposure (21). The Wells–Riley model and its deviations have been extensively employed in studies on the transmission of airborne infectious diseases, including COVID-19 (22). For instance, Wang et al. estimated the infection probability of COVID-19 for a 2-h fight (23) and for Chinese long-distance trains (24) under scenarios at various face mask efficiency levels. Che et al. (25) and Sha et al. (26) predicted the impact of ventilation systems on COVID-19 control and prevention in high-rise buildings.

Hence, in this study, we used the generalized SEIR model in conjunction with the Wells–Riley model to simulate the transmission of COVID-19 in sports stadiums under the scenarios of various preventive strategies, including vaccination, face mask wearing, and nucleic acid testing. We first used the generalized SEIR model developed by Cheynet (13) to simulate the impact of full vaccination delivery in the general population in China. We assumed that a part of the general population would become audience members of the CSL and that vaccination would decrease the number of infectious audience members entering the sports stadiums. Next, we used the Wells–Riley model, involving face mask efficiency (22) and nucleic acid testing, to simulate the transmission of COVID-19 among the audience members under scenarios including (1) without any preventive strategy, (2) with vaccination against COVID-19, (3) with a negative nucleic acid testing result within 7 days before admission, (4) with combined vaccination and nucleic acid testing, (5) with combined nucleic acid testing and face mask wearing, and (6) with combined vaccination, nucleic acid testing, and face mask wearing. By combining those two mathematic models, this study involved the effect of vaccination policy on the general population in a sports stadium and visualized the effects of face mask wearing and nucleic acid testing on COVID-19 transmission in CLS audiences. The study findings indicate the potential infection risk in sports stadium, suggest some effective measures for infection control in the post-pandemic era, and provide some evidence for policymakers to reopen large sports events.

## Methods

### Generalized SEIR model and fitting

To characterize the epidemic of COVID-19 during the 2021 CSL game season, we used the generalized SEIR model with seven different states, namely, susceptible (*S*), insusceptible (*P*), exposed (*E*, in a latent period, infected but not showing infectiousness), infectious (*I*, infectious and not yet quarantined), quarantined (Q, confirmed), recovered (*R*), and death (*D*). *S(t), P(t), E(t), I(t), Q(t), R(t)*, and *D(t)* denote, at time *t*, the number of susceptible, insusceptible, exposed, infectious, quarantined, recovered, and death cases, respectively. Their relations are governed by a serial of equations, which can be formulated through ordinary differential equations (ODEs) as follows (13):


(1) $$\begin{array}{l} \;dS(t)/dt=-\beta (t)I(t)S(t)/N-\alpha \;S(t),\\ \;dP(t)/dt=\;\alpha \;S(t),\\ \;dE(t)/dt=\beta (t)I(t)S(t)/N-\;\gamma (t)E(t),\\ \;dI(t)/dt=\gamma (t)E(t)-\delta (t)I(t),\\ dQ(t)/dt=\delta \left(t\right)I\left(t\right)-\lambda \left(t\right)Q\left(t\right)-\;\kappa (t)Q(t),\\ \;dR(t)/dt=\lambda \left(t\right)Q\left(t\right)\text{​},\\ \;dD(t)/dt=\kappa (t)Q(t), \end{array}$$

where *N* is the total population and the coefficients α, β, γ^−1^, δ^−1^, λ*(t)*, and κ*(t)* are the protection rate, infection rate, average latent time, average quarantine time, cure rate, and mortality rate, respectively. We assumed that *N* = *S*+*P*+*E*+*I*+*Q*+*R*+*D* is constant and the model ignores immigration, emigration, birth, and death unrelated to COVID-19; that the infectious population is evenly mixed with the susceptible population; that the people in the model have the same odds of making a decision about watching a game in a stadium.

The seven states {*S(t), P(t), E(t), I(t), R(t), D(t)*}2 with fitted parameters {α, β, γ^−1^, δ^−1^, λ*(t)*, κ*(t)*} were calculated by a non-linear least-square function based on Chinese data from 20 April to 10 September. The ODEs were written in a matrix form and solved using the classic fourth-order Runge–Kutta method to find the time evolution of the seven states. The matrix form is shown as follows:


(2) $$\begin{array}{l} \text{dY/dt=G}\times \text{Y+F} \end{array}$$

where


(3) $$\begin{array}{l} Y={\left[S,E,I,Q,R,D,P\right]}^{T} \end{array}$$

(4) $$G=\left(\begin{array}{ccccccc} \alpha & 0 & 0 & 0 & 0 & 0 & 0\\ 0 & -\alpha & 0 & 0 & 0 & 0 & 0\\ 0 & \alpha & -\delta & 0 & 0 & 0 & 0\\ 0 & 0 & \delta & -\kappa (t)-\lambda (t) & 0 & 0 & 0\\ 0 & 0 & 0 & \lambda (t) & 0 & 0 & 0\\ 0 & 0 & 0 & \kappa (t) & 0 & 0 & 0\\ 0 & 0 & 0 & 0 & 0 & 0 & 0 \end{array}\right)$$

(5) $$\begin{array}{l} F=S(t)\times I(t)\times \left(\begin{array}{c} \;-\beta /N\\ \;\beta /N\\ \;0\\ \;0\\ \;0\\ \;0\\ \;0 \end{array}\right) \end{array}$$

The simulation time period was set from 20 April (the beginning of the 2021 game season, also the stationary phase of the COVID-19 pandemic in China) to 10 September (the endpoint of an outbreak in this time period). The 2021 game season finished on 5 August, which was included in this simulation time period. The initial numbers of the coefficients *N, R*_0_, *D*_0_, and *Q*_0_ were determined according to the daily briefing of the National Health Commission (NHC) of China (27), and *E*_0_ and *I*_0_ were inferred according to prior studies based on Chinese data (13). The values and sources of the coefficients can be seen in Table 1. For the optimization parameter, the “fit_SEIQRDP” function was used, which was available in the generalized SEIR model package developed by Cheynet in MATLAB (13).

**Table 1:** Parameters in the SEIR model.

**Parameter**	**Value**	**Description and source**
*N*	26,418,000	Total population of cities where pandemic outbroke during the 2021 game season (41).
*R_0_*	85,612	Number of cumulative recovered cases on 20 April, 2021 reported by NHC (27).
*D_0_*	4,636	Number of cumulative dead cases on 20 April, 2021 reported by NHC (27).
*Q_0_*	616	Number of quarantined cases on 20 April, 2021 reported by NHC, including confirmed patients and asymptomatic infectors under medical observation (27).
*E_0_*	616	Equal to Q_0_ (13).
*I_0_*	123	1/5 of Q_0_ (13).

The fitting is shown using the daily number of current quarantined *cases Q(t)* and cumulative recovered cases *R(t)*, which can be observed in the real world and was reported in the daily briefing from the NHC. Susceptible *S(t)*, insusceptible *P(t)*, exposed *E(t)*, and infectious *I(t)* cases and the parameters α, β, γ^−1^, δ^−1^, λ*(t)*, and κ*(t)* could also be computed by this package, while *I(t)* and α were used as targets.

The boundedness and non-negativity of the model variables can be seen in Supplementary materials.

### Integrating vaccination into the generalized SEIR model

We simulated the effectiveness of vaccination on *I*_v_ *(t)* by adding the efficacy of inactivated vaccines to α. In the vaccination scenario, people were assumed to have received two doses of inoculation and thus have completed the immunogenic process when entering the model, and the effect of vaccination was to increase the protection rate α and thereby decrease the susceptible population; although the vaccine may offer less protection due to the emergency of new variants and the immunity might wane over long timescales, we assumed that the efficacy of vaccination did not change in the simulation of the 4-month game season. The WHO report on the inactive Sinopharm/BBIBP COVID-19 vaccine showed that the overall efficacy of two doses of the vaccine is 0.78 (28). By definition, vaccine efficacy is the proportional reduction in infection rates. Therefore, we assumed in this study that vaccination would increase α by 0.78. The equation can be written as:


(6) $$\begin{array}{l} \alpha_{v}=\alpha +0.78 \end{array}$$

*I(t)* and *I*_v_ *(t)* are the numbers of infectious people without and with vaccination in the general population, respectively, which were used as parameters in the next step of the infection simulation in the stadium.

### Wells–Riley model and infection probability in the stadium

According to the Wells–Riley equation, the risk of infection by pathogens of a susceptible person through the air in a ventilated room is related to the quanta she or he inhales, the amount of pulmonary ventilation, the concentration of pathogen-bearing particles in the inhaled air, and the time of exposure. Based on these assumptions, the equation can be written as (21, 29):


(7) $$\begin{array}{l} p=1-exp\left(-\frac{Aqvt}{Q}\right)\;\left(7\right) \end{array}$$

where *p* is the infection probability, *A* is the number of infectious people entering the room, *q* is the quanta produced by an infected individual (quanta/h), *v* is the pulmonary ventilation rate of a person (m^3^/h), *t* is the exposure time (h), and *Q* is the outdoor air supply rate (m^3^/h). This equation is based on the assumptions that infectious particles are well-mixed in airspace, exposure to one quantum of infection provides an average probability of 63.2% based on a Poisson distribution, and the outdoor air supply rate *Q* remains constant.

Rudnick and Milton proposed a modified Wells–Riley model using the exhaled air volume fraction, which does not require the second assumption (30). *Q* could be a function of the exhaled air volume fraction, the number of people, and the pulmonary ventilation rate *v*; the equation can be written as:


(8) $$\begin{array}{l} Q=nv/\bar{f} \end{array}$$

where $\bar{f}$ is the exhaled air volume fraction and *n* is the number of people in the ventilated room (30). Sheng (31) considered that the volume of a sports stadium would affect the transmission of pathogens, and the audience could use personal respiratory protection to decrease the probability of infection. Therefore, Sheng combined the modified Wells–Riley model by Fennelly and Nardell (32), which included personal respiratory protection in the equation, and wrote the equation as (31):


(9) $$\begin{array}{l} B=1-exp\left\{\left(-\frac{\bar{f}Aqt\theta }{n}\right)\left[1-\exp\left(-\frac{nvt}{V\bar{f}}\right)\right]\right\} \end{array}$$

where θ is the penetration ratio of the protective respirator, which reflects the amount of leakage through and around the respirator (32). For instance, θ is 0.25 for an ordinary surgical mask (26), and θ is 1 without any respirator (32); *V* is the volume of the stadium.

The value of quantum varies significantly in different studies, as it depends on the types of pathogens and the estimation methods used (26). Sheng asserted that the quantum in a sports stadium is influenced by *A*, the number of infectious people entering the stadium, and assumed that the quantum has a linear relationship with *A* (31). According to Sheng's method, the equation can be written as:


(10) $$\begin{array}{l} q=0.16A+36 \end{array}$$

where *A* is estimated based on *I(t)* on each match day according to the results of the SEIR model and the false negative rate of nucleic acid testing. For instance, *A* is equal to *0.3nI(t)/N* when the audience are required to show a negative nucleic acid testing result with a 30% false negative rate.

The parameters of the six scenarios used in the Wells–Riley model are shown in Table 2.

**Table 2:** Parameters in the Wells–Riley model.

**Parameter**	**Description**	**S1**	**S2**	**S3**	**S4**	**S5**	**S6**
*A*	Number of infectious people entering the stadium	nI(t)/N	nI_v_(t)/N	0.3nI(t)/N	0.3nI_v_ (t)/N	0.3nI (t)/N	0.3nI_v_ (t)/N
θ	Penetration ratio of face mask	1 (32)	1 (32)	1 (32)	1 (32)	0.25 (26)	0.25 (26)
*v*	Pulmonary ventilation rate of a person	3m^3^ h (31)
*n*	Number of people in the stadium	30000 (4)
*V*	Volume of the stadium	30000 m^3^ (31)
*t*	Exposure time	2 h
$\bar{f}$	Exhaled air volume fraction	1.1 m^3^ h (42)
*q*	Quanta produced by infectious people	0.16A+36 (31)

S1, Scenario 1, without any preventive strategy; S2, Scenario 2, with vaccination against COVID-19; S3, Scenario 3, with a negative nucleic acid testing result within 7 days before admission; S4, Scenario 4, with combined vaccination and nucleic acid testing; S5, Scenario 5, with combined nucleic acid testing and face mask wearing; S6, Scenario 6, with combined vaccination, nucleic acid testing, and face mask wearing.

## Results

### Simulation of infection in the general population with and without vaccination

**Figure 1:**
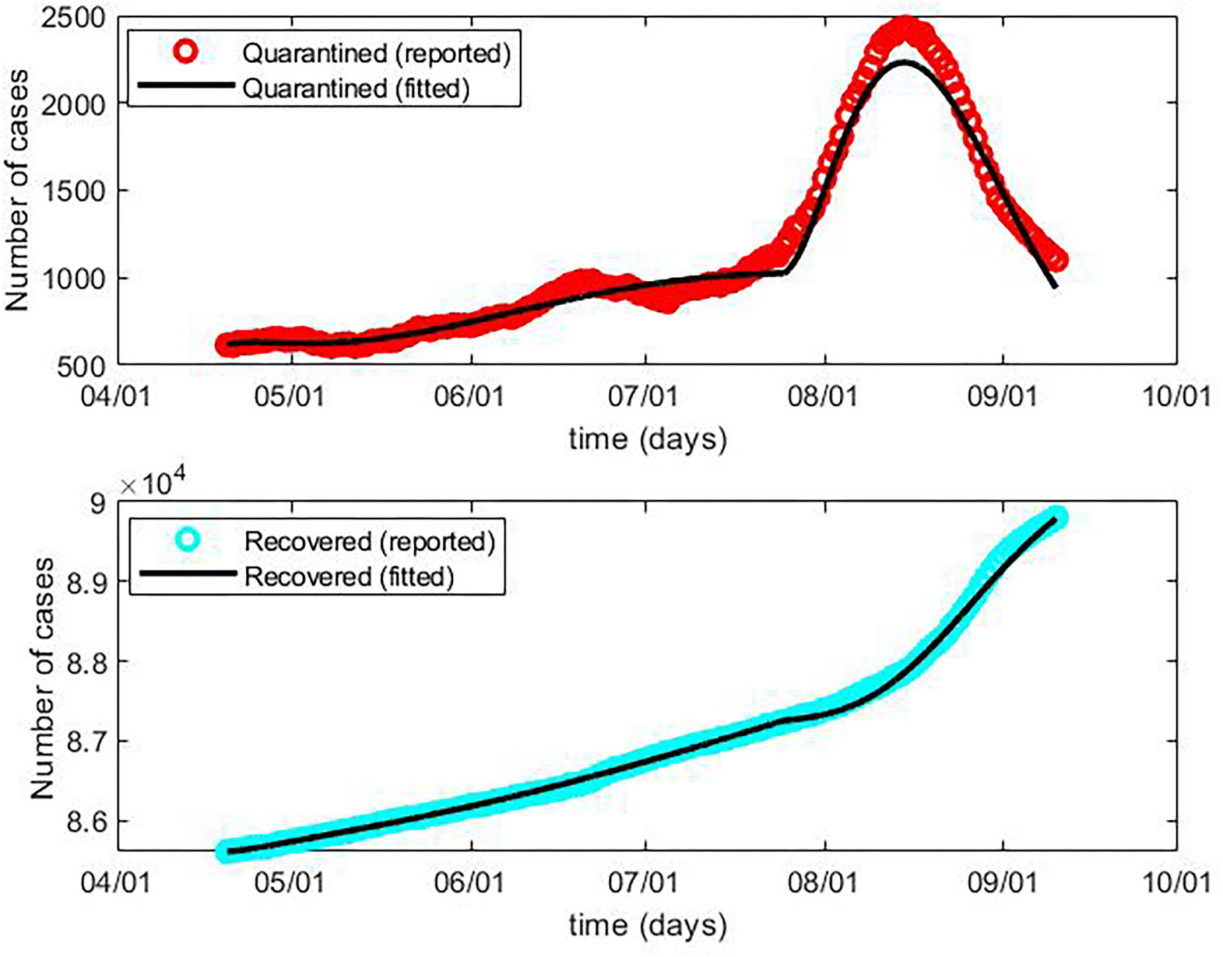
The reported and fitted current quarantined cases and cumulative recovered cases of COVID-19, China, 20 April to 10 September.

We applied the above-described SEIR model to fit the public data of the daily current quarantined and cumulative recovered cases from 20 April to 10 September (Figure 1). The epidemic curve during this period was divided into two phases. In the first phase from 20 April to 24 July, the number of quarantined cases was initially low and slowly increased. In the second phase from 25 July to 10 September, there was an outbreak of the pandemic, reaching a peak of over 2,000 quarantined cases in early August.

Through extensive simulations, the values for the unknown model parameters α, β, γ^−1^, δ^−1^, λ*(t)*, and κ*(t)* were calculated and can be seen in Table 3. The protection rate α in the first phase was 0.04, lower than that in the second phase (0.37), which indicates that before the outbreak, people might have paid less attention to self-protection, and most of the general population were susceptible to SARS-CoV-2. The higher infection rate β and longer latent time γ^−1^ in the first phase showed that people were more likely to be infected by the virus and that it was more difficult to detect in the first phase, which afterward led to the surge of confirmed cases in the second phase. The accessibility of medical treatment for COVID-19 cases in China has maintained a high level since the initial outbreak of the pandemic in early 2020. As a result, the quarantine time δ^−1^ was maintained at 9 days in both phases. In addition to the constant parameters α, β, γ^−1^, and δ^−1^, this study showed the function of the cure rate λ*(t)* and the mortality rate κ*(t)*, which are the choices of best approximation determined by the generalized SEIR model. The values of these parameters best interpreted the data by showing a perfect fit of the curves for quarantined and recovered cases, which can be observed in the real world.

**Table 3:** Values for unknown parameters in the fitted SEIR model.

**Parameter**	**Description**	**20 April to 24 July**	**25 July to 10 September**
α	Protection rate	0.04	0.37
β	Infection rate	0.81	0.29
*γ^−1^*	Average latent time (day)	87.0	6.9
*δ^−1^*	Average quarantine time (day)	9.3	9.3
*λ(t)*	Cure rate	0.02/{1+exp[−0.46*(t−0.78)]}	0.07/{1+exp[−0.09*(t−26.11)]}
*κ(t)*	Mortality rate	[10^(^−4)]*{exp[−0.06*(t-69350)]^2^}	[10^(^−3)]/{exp[0.69*(t−4.49)]+exp[−0.69*(t−4.49)]}
*α_*v*_*	Protection rate with vaccination	0.82 (28)^a^

^a^ αv = α+0.78, 0.78 is the efficacy of Sinopharm/BBIBP COVID−19 vaccine reported by WHO.

**Figure 2:**
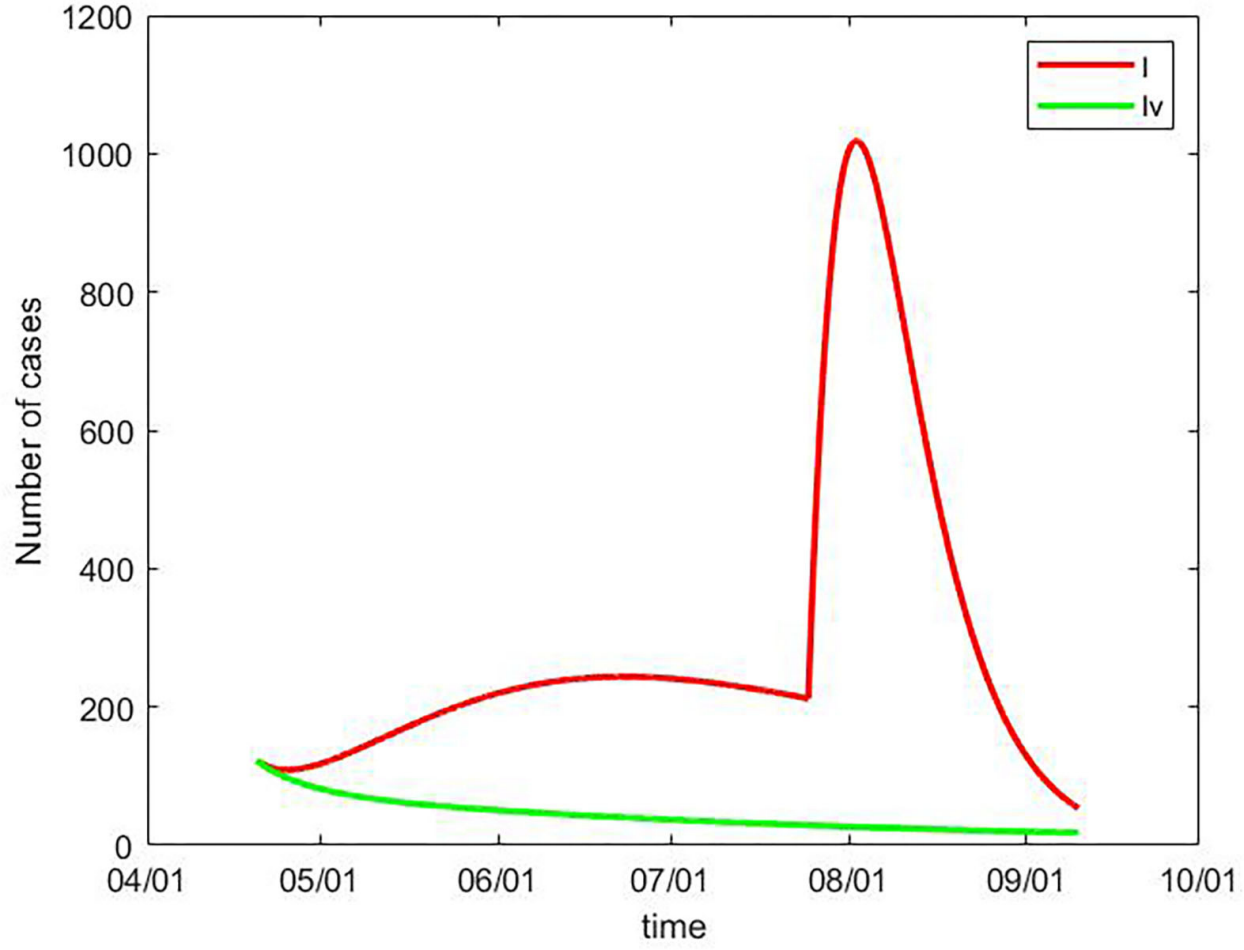
Number of infectious people with vaccination *I*_v_ *(t)* and without vaccination *I(t)*, China, 20 April to 10 September.

To understand the impact of vaccination, *I(t)*, the number of infectious people without vaccination was calculated. After adding the vaccine efficacy (0.78) to the initial protection rate, *I*_v_ *(t)*, the number of infectious people under vaccination was also calculated. *I(t)* and *I*_v_ *(t)* are shown in Figure 2, and the daily data from 20 April to 10 September can be seen in Supplementary Table S1. Without vaccination, the daily number of infectious people would have increased from 123 on 20 April to a peak of 1,018 on 7 August, and then decreased to 96 on 10 September. The total number of infectious cases would have been 43,455 during this time period. Under the assumption that the population were vaccinated, the number of infectious people would have constantly decreased from 123 on 20 April to 18 on 10 September. The outbreak of the pandemic in August, which has led to thousands of people becoming infected, could have been constantly controlled at a lower level. The total number of infectious cases during this time period would have been 6,417, which is 85.2% < the number without vaccination.

### Simulation of infection in the stadium audiences during the CSL game season

**Figure 3:**
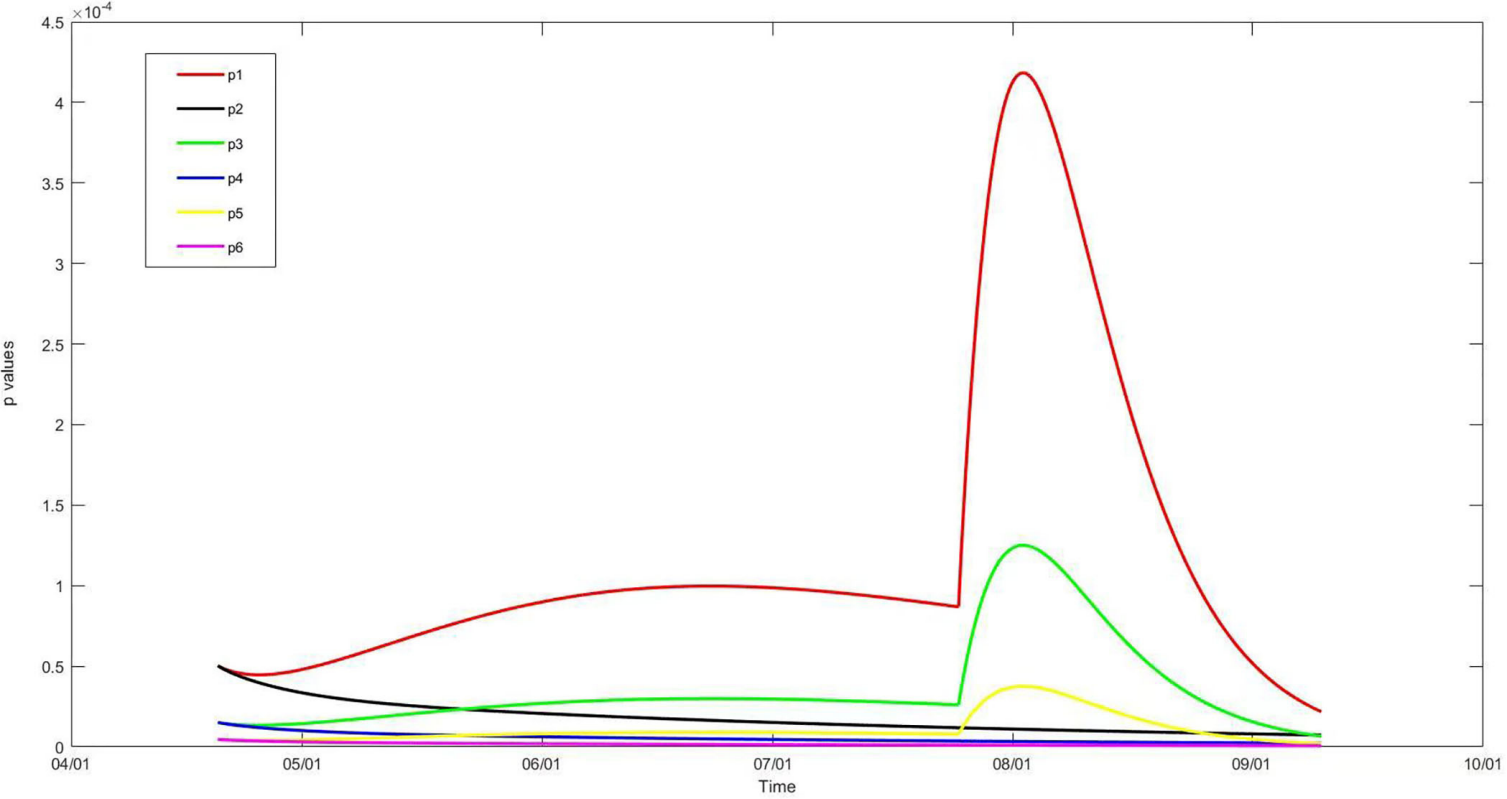
Infection probability (p) in stadium, 20 April to 10 September. p1, without any preventive strategy; p2, with vaccination against COVID-19; p3, with a negative nucleic acid testing result within 7 days before admission; p4, with combined vaccination and nucleic acid testing; p5, with combined nucleic acid testing and face mask wearing; p6, with combined vaccination, nucleic acid testing, and face mask wearing.

Based on the number of infectious people calculated by the generalized SEIR model, parameter *A* in the Wells–Riley model with and without vaccination could be identified, and the daily infection probability of the stadium audiences during the 2021 CSL game season under six scenarios was calculated (Figure 3). The infection probabilities of Scenario 1 (p1), p3, and p5 reflect those scenarios without vaccination, and their curves were similar to that of *I(t)*. Meanwhile, the infection probabilities of Scenario 2 (p2), p4, and p6 reflect those scenarios with vaccination, which decreased progressively from 20 April to 10 September in accordance with the curve of *I*_v_ *(t)*.

According to the game schedule, there were 116 games held on 58 game days in the 2021 season. The daily and the total number of infected people for the game season are shown in Table 4. If all of the 30,000 seats in the stadium were taken by audience members, just as it was before the COVID-19 pandemic, 3–25 audience members would have been infected with COVID-19 for each game day without any preventive strategies, and a total of 371 audiences would have been infected during this game season (Scenario 1). With the requirement of a negative nucleic acid test of SARS-CoV-2 within 7 days before admission, the daily number of infected audience members would have ranged from one to seven, and the total number of infected people during this game season would have been 118 (Scenario 3). The combination of nucleic acid testing and face mask wearing would have further decreased this number to 34, meaning 27 game days would be “completely safe” for the audiences and one or two audience members would have been infected for each of the other 31 game days (Scenario 5). If the general population were vaccinated before the game season began, the number of infected audiences would have been 79 during this season, with one to three audience members would have been infected on each game day (Scenario 2). The combination of vaccination and nucleic acid testing would have decreased the number of infected audience members to 14, with 42 safe game days (Scenario 4). The number of infected people would have been zero after rounding off when combining all three preventive strategies (Scenario 6).

**Table 4:** Number of infected audiences during 2021 CLS game season.

**Date**	**Number of infected spectators**					
	**S1**	**S2**	**S3**	**S4**	**S5**	**S6**
20 April	3	3	1	1	0	0
21 April	3	3	1	1	0	0
22 April	3	3	1	1	0	0
23 April	3	3	1	1	0	0
26 April	3	2	1	1	0	0
27 April	3	2	1	1	0	0
28 April	3	2	1	1	0	0
29 April	3	2	1	1	0	0
2 May	3	2	1	1	0	0
3 May	3	2	1	1	0	0
4 May	3	2	1	1	0	0
5 May	3	2	1	1	0	0
8 May	3	2	1	1	0	0
9 May	4	2	1	1	0	0
10 May	4	2	1	0	0	0
11 May	4	2	1	0	0	0
14 May	4	2	1	0	0	0
15 May	4	2	1	0	0	0
16 May	4	2	1	0	0	0
17 May	4	1	1	0	0	0
21 June	6	1	2	0	1	0
22 June	6	1	2	0	1	0
23 June	6	1	2	0	1	0
24 June	6	1	2	0	1	0
26 June	6	1	2	0	1	0
27 June	6	1	2	0	1	0
28 June	6	1	2	0	1	0
29 June	6	1	2	0	1	0
2 July	6	1	2	0	1	0
3 July	6	1	2	0	1	0
4 July	6	1	2	0	1	0
5 July	6	1	2	0	1	0
7 July	6	1	2	0	1	0
8 July	6	1	2	0	1	0
9 July	6	1	2	0	1	0
10 July	6	1	2	0	1	0
12 July	6	1	2	0	1	0
13 July	6	1	2	0	1	0
14 July	6	1	2	0	1	0
15 July	6	1	2	0	1	0
17 July	6	1	2	0	1	0
18 July	6	1	2	0	1	0
19 July	6	1	2	0	0	0
20 July	5	1	2	0	0	0
23 July	5	1	2	0	0	0
24 July	5	1	2	0	0	0
25 July	5	1	2	0	0	0
26 July	5	1	2	0	0	0
28 July	5	1	2	0	0	0
29 July	10	1	3	0	1	0
30 July	14	1	4	0	1	0
31 July	17	1	5	0	2	0
2 August	22	1	7	0	2	0
3 August	24	1	7	0	2	0
4 August	24	1	7	0	2	0
5 August	25	1	7	0	2	0
Total	371	79	118	14	34	0

S1, Scenario 1, without any preventive strategy; S2, Scenario 2, with vaccination against COVID−19; S3, Scenario 3, with a negative nucleic acid testing result within 7 days before admission; S4, Scenario 4, with combined vaccination and nucleic acid testing; S5, Scenario 5, with combined nucleic acid testing and face mask wearing; S6, Scenario 6, with combined vaccination, nucleic acid testing, and face mask wearing.

The daily infection probability and the number of infected audiences can be seen in Supplementary Table S1.

## Discussion

We conducted mathematic modeling analyses to assess the effect of vaccination, nucleic acid testing, and face mask wearing on the transmission of COVID-19 in stadium audiences during the 2021 CSL game season. By using these modeling approaches, this study incorporated the natural history of COVID-19 infection and predicted the exact number of infected audience members upon using various preventive strategies, which may guide the resumption of large-scale sports events with audiences.

The generalized SEIR model interpreted the dynamics of COVID-19 transmission over 5 months, from a relatively stationary phase to an outbreak of cases. The values of the parameters in this study share some similarities with those in previous studies. The protection rate was 0.04 in the first phase, which is close to Cheynet's estimate of 0.06 (13), and a little bit less than the estimate of 0.17 by Peng et al. (33). These two studies used the generalized SEIR model to simulate Chinese data from January 2020 onward. The scatter outbreak in the first phase may have alerted people and the government to conduct some preventive interventions (33), and led to an increase in the protection rate to 0.37 in the later outbreak from 25 July to 10 September. The infection rate was 0.81 in the first phase, which is equal to the estimate in Cheynet's study (13) and close to the estimate of 1.0 in Peng et al.'s study (33), which indicates that most people were susceptible to the virus at the outbreak of COVID-19. As the protection rate increased, the infection rate decreased to 0.29 in the second phase. According to the clinical study of Jiang et al. on the characteristics of COVID-19 cases, the average latent time is 5.2 days, and 95% of cases develop clinical symptoms in 12.5 days (2), which is similar to the latent time of 6.9 days in the later phase in our study. The latent time of 6.9 days in the second phase and the quarantine time of 9.3 days estimated in this study are also close to the estimates of 5 and 10 days in Cheynet's study, and of 2 and 6.6 days in Peng et al.'s study, respectively. Simulating the COVID-19 pandemic in different time periods may lead to minor variants in parameter estimation. However, the latent time of 87 days for the first phase was much longer than the latent time shown in previous studies (2, 13, 33). This extensive simulation tried to fit the real-world data in a stationary stage by extending the latent time and, therefore, showed a flat curve of quarantined cases. This indicates that less outbreaks scattered across the country may be difficult to predict using the SEIR model. This mathematical model is more useful for interpreting a typical pandemic with a high peak value.

As the SARS-CoV-2 variants become more transmissible, the introduction of SARS-CoV-2 in audiences in sports stadiums is almost inevitable (34). The resumption of sports events with audiences, which is undoubtedly important for the audience experience and the development of the sports industry, can greatly increase the risk of infection due to mass gathering and transportation (35). In this study, we showed that without any preventive strategies, over 300 audience members would have been infected in the 2021 game season. For the sake of the COVID-19 pandemic, the 2021 game schedule was simplified by cutting 50% of games, and this season ended with a premature closing before the major outbreak in August. It can be inferred that more audience members would have been infected if home-and-away games were played all over the country.

The combined use of all three strategies, namely, vaccination, nucleic acid testing, and face mask wearing, was the most effective measure in this study, which would have completely protected audiences from infection. Vaccination can effectively control the transmission of SARS-CoV-2 in the general population and audiences. By simulating a scenario with vaccination, this study showed that high levels of population immunity would be generated if all members of the general population received vaccination with 78% efficiency. As a result, the number of infectious people entering stadiums could be decreased, further leading to a lower infection probability of audience members. Moreover, vaccination was shown to be more effective than nucleic acid testing when independently using either strategies. Given that watching a game while wearing a face mask impairs the audience experience, vaccination and nucleic acid testing may work as a compromise between safety and the audience experience when designing preventive strategies for a sports event.

However, the effectiveness of vaccination against COVID-19 is challenged by some uncertainties. First, the vaccine efficacy has been shown to range from 22% (36) to 100% (37) in clinical trials of different types of vaccines. Second, the Omicron variant significantly increased the transmissibility and immune evasion of the virus. Its influence on vaccine efficacy still holds a great degree of ambiguity (38), and booster doses may be needed consistently to resist the waning of vaccine efficacy over time (39). Moreover, the vaccine mistrust and anti-vaccine movement in some places are a threat to the coverage of vaccination (40). Given the uncertainties around vaccination strategy, this study also simulated some scenarios in which there was no vaccination. The results showed that the independent use of nucleic acid testing would have decreased the number of infected audience members by two-third, and the combined use of nucleic acid testing and face mask wearing would have further decreased the number of infected audience members. Although nucleic acid testing and face mask wearing are less effective than strategies combined with vaccination, policymakers can consider these strategies to control the infection probability to some degree.

There are several limitations related to modeling assumptions, data limitations, and uncertainty. First, modeling is a process to simplify a real situation. During the 2021 CSL game season, the extensive COVID-19 pandemic that broke out in late 2019 had been controlled and limited to scattered outbreaks in a couple of cities. Home-and-away games were also limited in two cities. Therefore, some assumptions of the SEIR model, including an even mix of the infectious and susceptible populations and people having the same odds ratio of entering the stadium, may not be well fitted to a real-world situation. However, most of the parameter estimates in our study were reasonable and in accordance with those of previous studies based on Chinese data (2, 13, 33). It was indicated that the generalized SEIR model and the initial conditions used in this study are efficient for the prediction of COVID-19 transmission in China. Second, the simulation results based on the Wells–Riley model cannot be validated by real-world data, because most of the 2021 CLS games were played in an empty stadium. Third, there is uncertainty around some of the model parameters, such as the vaccine efficacy, penetration ratio of face mask, and false negative ratio of nucleic acid testing, which were extracted according to previous studies. Future modeling studies could build on this effort when updated data become available for model parameter estimation and calibration targets. The results of the Wells–Riley model could be tested when the auditorium reopening policy was implemented for large sports events in China.

In conclusion, the combined use of the SEIR and Wells–Riley models offers a tool to benchmark the effects of preventive strategies against COVID-19 in activities involving mass gatherings such as large sports events, which include transportation and confined-space gathering. Our findings showed that the reopening of the auditorium would have a potential infection risk without any preventive strategies. The combined use of vaccination, nucleic acid testing, and face mask wearing could effectively protect audience members against infection. The use of any two of these strategies could significantly lower the infection rates. Accordingly, the public can understand the risk of game watching, and policymakers can consider the combined use of preventive strategies when holding sports events, which could create a balance between audience experiences and COVID-19 infection control.

## Data availability statement

The raw data supporting the conclusions of this article will be made available by the authors, without undue reservation.

## Author contributions

HZ, ZL, and ND conceptualized the study. XS, HR, XH, WZ, and CL collated the data. ZL and XS analyzed the data. HZ drafted the initial manuscript. ZL and ND reviewed and revised the manuscript. All authors contributed to the interpretation of the results and approved the submitted version.

## Funding

This study was supported by the Fundamental Research Funds for the Central Universities, Beijing Sport University (Grant Nos. 2022YB007, 2021TD002, and 3001049).

## Conflict of interest

The authors declare that the research was conducted in the absence of any commercial or financial relationships that could be construed as a potential conflict of interest.

## Publisher's note

All claims expressed in this article are solely those of the authors and do not necessarily represent those of their affiliated organizations, or those of the publisher, the editors and the reviewers. Any product that may be evaluated in this article, or claim that may be made by its manufacturer, is not guaranteed or endorsed by the publisher.
