# Supplementary material for: Modeling the effects of vaccination, nucleic acid testing, and face mask wearing interventions against COVID-19 in large sports events
Source: Front Public Health. 2022 Nov 9;10:1009152. doi: 10.3389/fpubh.2022.1009152 (PMC9682230; doi:10.3389/fpubh.2022.1009152)
Supplement: Supplementary file 1 [file Table_1.DOCX]

Supplementary Material

# The boundedness and nonnegativity of parameters for the generalized SEIR model.

The generalized SEIR model we used in this study is following the one used by Cheynet [1], which was derived from Feng and Thieme’s study [2]. Total population N divide into seven states: susceptible (*S*), insusceptible (*P*), exposed (*E*, in a latent period, infected but not showing infectiousness), infectious (*I*, infectious and not yet quarantined), quarantined (Q, confirmed), recovered (*R*), and death (*D*). Their relations are shown in Supplementary Figure S1 and governed by an equation system, which can be formulated through ordinary differential equations (ODEs) as follows [1]:

$dS(t)/dt=-\beta(t)I(t)S(t)/N-\alpha S(t)$*,*

$dP(t)/dt=\alpha S(t)$*,*

$dE(t)/dt=\beta(t)I(t)S(t)/N-\gamma(t)E(t)$*,*

$dI(t)/dt=\gamma(t)E(t)-\delta(t)I(t)$*,*  (1)

$dQ(t)/dt=\delta\left( t \right)I\left( t \right)-\lambda\left( t \right)Q\left( t \right)-\kappa(t)Q(t)$*,*

$dR(t)/dt=\lambda\left( t \right)Q\left( t \right)$*,*

$dD(t)/dt=\kappa(t)Q(t)$*,*

where *N* is the total population, and *S(t), P(t), E(t), I(t), Q(t), R(t),* and *D(t)* denote, at time *t*, the number of susceptible, insusceptible, exposed, infectious, quarantined, recovered, and death cases, respectively. The coefficients *α, β, γ^-1^, δ^-1^, λ(t),* and *κ(t)* are the protection rate, infection rate, average latent time, average quarantine time, cure rate, and mortality rate, respectively. We note that the parameters of the model are non-negative, and N = S+P+E+I+Q+R+D is constant. According to Theorem 2.1 proved by Feng and Thieme’s study [2], there should exist a unique solution to the system (1), and the solution is non-negative and bounded for any given initial data *(R_0,_ D_0,_ Q_0,_ E_0,_ I_0_)* ≥ 0*.*


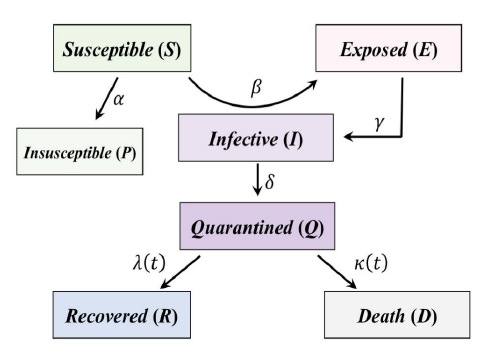


**Supplementary Figure S1.** Schematic diagram of the generalized SEIR model. *This picture is cited from Peng et al.’s study [3].*

Based on the generalized SEIR model, number of infectious people *I* with and without vaccination was calculated. The daily value of *I(t)* and *I*_v_ *(t)* are shown in Supplementary Table S1.

# Infection in stadium audience during CSL game season

based on the numbers of infectious people calculated by the generalized SEIR model, parameter *A* in the Wells-Riley model with and without vaccination could be identified, and the infection probability and number of infected audiences for each day from 20 April to 10 September are shown in Supplementary Table S1.

**Reference**

1. Cheynet E. Generalized SEIR Epidemic Model (fitting and computation). 2020.doi:doi:10.5281/ZENODO.3911854.

2. Feng Z, Thieme HR. Recurrent outbreaks of childhood diseases revisited: the impact of isolation. Math biosci 1995;128(1-2):93-130

3. Peng L, Yang W, Zhang D, et al. Epidemic analysis of COVID-19 in China by dynamical modeling. medRxiv.

**Supplementary Table S1.** Number of infectious people in the general population, infection probability and number of infected audiences in the sports stadium.

| Date | I(t) | I_v_(t) | Infection probability in stadium | | | | | | Number of infected spectators for each game | | | | | | Number of games |
| --- | --- | --- | --- | --- | --- | --- | --- | --- | --- | --- | --- | --- | --- | --- | --- |
|  |  |  | S1 | S2 | S3 | S4 | S5 | S6 | S1 | S2 | S3 | S4 | S5 | S6 |  |
| 20 April | 123 | 123 | 4.80E-05 | 4.80E-05 | 1.40E-05 | 1.40E-05 | 4.30E-06 | 4.30E-06 | 1.4 | 1.4 | 0.4 | 0.4 | 0.1 | 0.1 | 2 |
| 21 April | 118 | 117 | 4.70E-05 | 4.60E-05 | 1.40E-05 | 1.40E-05 | 4.20E-06 | 4.10E-06 | 1.4 | 1.4 | 0.4 | 0.4 | 0.1 | 0.1 | 2 |
| 22 April | 114 | 111 | 4.60E-05 | 4.40E-05 | 1.40E-05 | 1.30E-05 | 4.10E-06 | 3.90E-06 | 1.4 | 1.3 | 0.4 | 0.4 | 0.1 | 0.1 | 2 |
| 23 April | 112 | 107 | 4.50E-05 | 4.20E-05 | 1.30E-05 | 1.30E-05 | 4.00E-06 | 3.80E-06 | 1.4 | 1.3 | 0.4 | 0.4 | 0.1 | 0.1 | 2 |
| 24 April | 110 | 103 | 4.50E-05 | 4.10E-05 | 1.30E-05 | 1.20E-05 | 4.00E-06 | 3.70E-06 | 1.3 | 1.2 | 0.4 | 0.4 | 0.1 | 0.1 | 0 |
| 25 April | 109 | 99 | 4.50E-05 | 3.90E-05 | 1.30E-05 | 1.20E-05 | 4.00E-06 | 3.50E-06 | 1.3 | 1.2 | 0.4 | 0.4 | 0.1 | 0.1 | 0 |
| 26 April | 109 | 96 | 4.50E-05 | 3.80E-05 | 1.30E-05 | 1.10E-05 | 4.00E-06 | 3.40E-06 | 1.3 | 1.1 | 0.4 | 0.3 | 0.1 | 0.1 | 2 |
| 27 April | 110 | 93 | 4.50E-05 | 3.70E-05 | 1.40E-05 | 1.10E-05 | 4.10E-06 | 3.30E-06 | 1.4 | 1.1 | 0.4 | 0.3 | 0.1 | 0.1 | 2 |
| 28 April | 111 | 90 | 4.60E-05 | 3.60E-05 | 1.40E-05 | 1.10E-05 | 4.10E-06 | 3.20E-06 | 1.4 | 1.1 | 0.4 | 0.3 | 0.1 | 0.1 | 2 |
| 29 April | 112 | 87 | 4.70E-05 | 3.50E-05 | 1.40E-05 | 1.00E-05 | 4.20E-06 | 3.10E-06 | 1.4 | 1.0 | 0.4 | 0.3 | 0.1 | 0.1 | 2 |
| 30 April | 114 | 85 | 4.80E-05 | 3.40E-05 | 1.40E-05 | 1.00E-05 | 4.30E-06 | 3.00E-06 | 1.4 | 1.0 | 0.4 | 0.3 | 0.1 | 0.1 | 0 |
| 1 May | 116 | 83 | 4.90E-05 | 3.30E-05 | 1.50E-05 | 9.90E-06 | 4.40E-06 | 3.00E-06 | 1.5 | 1.0 | 0.4 | 0.3 | 0.1 | 0.1 | 0 |
| 2 May | 119 | 81 | 5.00E-05 | 3.20E-05 | 1.50E-05 | 9.70E-06 | 4.50E-06 | 2.90E-06 | 1.5 | 1.0 | 0.4 | 0.3 | 0.1 | 0.1 | 2 |
| 3 May | 122 | 79 | 5.10E-05 | 3.20E-05 | 1.50E-05 | 9.50E-06 | 4.60E-06 | 2.80E-06 | 1.5 | 0.9 | 0.5 | 0.3 | 0.1 | 0.1 | 2 |
| 4 May | 125 | 77 | 5.20E-05 | 3.10E-05 | 1.60E-05 | 9.20E-06 | 4.70E-06 | 2.80E-06 | 1.6 | 0.9 | 0.5 | 0.3 | 0.1 | 0.1 | 2 |
| 5 May | 128 | 75 | 5.40E-05 | 3.00E-05 | 1.60E-05 | 9.10E-06 | 4.80E-06 | 2.70E-06 | 1.6 | 0.9 | 0.5 | 0.3 | 0.1 | 0.1 | 2 |
| 6 May | 131 | 74 | 5.50E-05 | 3.00E-05 | 1.60E-05 | 8.90E-06 | 4.90E-06 | 2.70E-06 | 1.6 | 0.9 | 0.5 | 0.3 | 0.1 | 0.1 | 0 |
| 7 May | 134 | 72 | 5.60E-05 | 2.90E-05 | 1.70E-05 | 8.70E-06 | 5.10E-06 | 2.60E-06 | 1.7 | 0.9 | 0.5 | 0.3 | 0.2 | 0.1 | 0 |
| 8 May | 138 | 71 | 5.80E-05 | 2.90E-05 | 1.70E-05 | 8.60E-06 | 5.20E-06 | 2.60E-06 | 1.7 | 0.9 | 0.5 | 0.3 | 0.2 | 0.1 | 2 |
| 9 May | 141 | 70 | 5.90E-05 | 2.80E-05 | 1.80E-05 | 8.40E-06 | 5.30E-06 | 2.50E-06 | 1.8 | 0.8 | 0.5 | 0.3 | 0.2 | 0.1 | 2 |
| 10 May | 145 | 69 | 6.10E-05 | 2.80E-05 | 1.80E-05 | 8.30E-06 | 5.50E-06 | 2.50E-06 | 1.8 | 0.8 | 0.5 | 0.2 | 0.2 | 0.1 | 2 |
| 11 May | 148 | 67 | 6.20E-05 | 2.70E-05 | 1.90E-05 | 8.10E-06 | 5.60E-06 | 2.40E-06 | 1.9 | 0.8 | 0.6 | 0.2 | 0.2 | 0.1 | 2 |
| 12 May | 152 | 66 | 6.40E-05 | 2.70E-05 | 1.90E-05 | 8.00E-06 | 5.70E-06 | 2.40E-06 | 1.9 | 0.8 | 0.6 | 0.2 | 0.2 | 0.1 | 0 |
| 13 May | 155 | 65 | 6.50E-05 | 2.60E-05 | 2.00E-05 | 7.90E-06 | 5.90E-06 | 2.40E-06 | 2.0 | 0.8 | 0.6 | 0.2 | 0.2 | 0.1 | 0 |
| 14 May | 159 | 64 | 6.70E-05 | 2.60E-05 | 2.00E-05 | 7.80E-06 | 6.00E-06 | 2.30E-06 | 2.0 | 0.8 | 0.6 | 0.2 | 0.2 | 0.1 | 2 |
| 15 May | 163 | 63 | 6.80E-05 | 2.60E-05 | 2.00E-05 | 7.70E-06 | 6.10E-06 | 2.30E-06 | 2.0 | 0.8 | 0.6 | 0.2 | 0.2 | 0.1 | 2 |
| 16 May | 166 | 62 | 6.90E-05 | 2.50E-05 | 2.10E-05 | 7.60E-06 | 6.20E-06 | 2.30E-06 | 2.1 | 0.8 | 0.6 | 0.2 | 0.2 | 0.1 | 2 |
| 17 May | 170 | 62 | 7.10E-05 | 2.50E-05 | 2.10E-05 | 7.50E-06 | 6.40E-06 | 2.20E-06 | 2.1 | 0.7 | 0.6 | 0.2 | 0.2 | 0.1 | 2 |
| 18 May | 173 | 61 | 7.20E-05 | 2.50E-05 | 2.20E-05 | 7.40E-06 | 6.50E-06 | 2.20E-06 | 2.2 | 0.7 | 0.7 | 0.2 | 0.2 | 0.1 | 0 |
| 19 May | 177 | 60 | 7.40E-05 | 2.40E-05 | 2.20E-05 | 7.30E-06 | 6.60E-06 | 2.20E-06 | 2.2 | 0.7 | 0.7 | 0.2 | 0.2 | 0.1 | 0 |
| 20 May | 180 | 59 | 7.50E-05 | 2.40E-05 | 2.30E-05 | 7.20E-06 | 6.80E-06 | 2.20E-06 | 2.3 | 0.7 | 0.7 | 0.2 | 0.2 | 0.1 | 0 |
| 21 May | 184 | 58 | 7.60E-05 | 2.40E-05 | 2.30E-05 | 7.10E-06 | 6.90E-06 | 2.10E-06 | 2.3 | 0.7 | 0.7 | 0.2 | 0.2 | 0.1 | 0 |
| 22 May | 187 | 58 | 7.80E-05 | 2.30E-05 | 2.30E-05 | 7.00E-06 | 7.00E-06 | 2.10E-06 | 2.3 | 0.7 | 0.7 | 0.2 | 0.2 | 0.1 | 0 |
| 23 May | 190 | 57 | 7.90E-05 | 2.30E-05 | 2.40E-05 | 6.90E-06 | 7.10E-06 | 2.10E-06 | 2.4 | 0.7 | 0.7 | 0.2 | 0.2 | 0.1 | 0 |
| 24 May | 193 | 56 | 8.00E-05 | 2.30E-05 | 2.40E-05 | 6.80E-06 | 7.20E-06 | 2.10E-06 | 2.4 | 0.7 | 0.7 | 0.2 | 0.2 | 0.1 | 0 |
| 25 May | 196 | 56 | 8.20E-05 | 2.30E-05 | 2.40E-05 | 6.80E-06 | 7.30E-06 | 2.00E-06 | 2.4 | 0.7 | 0.7 | 0.2 | 0.2 | 0.1 | 0 |
| 26 May | 199 | 55 | 8.30E-05 | 2.20E-05 | 2.50E-05 | 6.70E-06 | 7.40E-06 | 2.00E-06 | 2.5 | 0.7 | 0.7 | 0.2 | 0.2 | 0.1 | 0 |
| 27 May | 202 | 54 | 8.40E-05 | 2.20E-05 | 2.50E-05 | 6.60E-06 | 7.50E-06 | 2.00E-06 | 2.5 | 0.7 | 0.8 | 0.2 | 0.2 | 0.1 | 0 |
| 28 May | 205 | 54 | 8.50E-05 | 2.20E-05 | 2.50E-05 | 6.50E-06 | 7.60E-06 | 2.00E-06 | 2.6 | 0.7 | 0.8 | 0.2 | 0.2 | 0.1 | 0 |
| 29 May | 208 | 53 | 8.60E-05 | 2.20E-05 | 2.60E-05 | 6.50E-06 | 7.70E-06 | 1.90E-06 | 2.6 | 0.6 | 0.8 | 0.2 | 0.2 | 0.1 | 0 |
| 30 May | 211 | 53 | 8.70E-05 | 2.10E-05 | 2.60E-05 | 6.40E-06 | 7.80E-06 | 1.90E-06 | 2.6 | 0.6 | 0.8 | 0.2 | 0.2 | 0.1 | 0 |
| 31 May | 213 | 52 | 8.80E-05 | 2.10E-05 | 2.60E-05 | 6.30E-06 | 7.90E-06 | 1.90E-06 | 2.6 | 0.6 | 0.8 | 0.2 | 0.2 | 0.1 | 0 |
| 1 June | 216 | 52 | 8.90E-05 | 2.10E-05 | 2.70E-05 | 6.30E-06 | 8.00E-06 | 1.90E-06 | 2.7 | 0.6 | 0.8 | 0.2 | 0.2 | 0.1 | 0 |
| 2 June | 218 | 51 | 9.00E-05 | 2.10E-05 | 2.70E-05 | 6.20E-06 | 8.10E-06 | 1.90E-06 | 2.7 | 0.6 | 0.8 | 0.2 | 0.2 | 0.1 | 0 |
| 3 June | 220 | 50 | 9.10E-05 | 2.00E-05 | 2.70E-05 | 6.10E-06 | 8.20E-06 | 1.80E-06 | 2.7 | 0.6 | 0.8 | 0.2 | 0.2 | 0.1 | 0 |
| 4 June | 222 | 50 | 9.20E-05 | 2.00E-05 | 2.80E-05 | 6.10E-06 | 8.30E-06 | 1.80E-06 | 2.8 | 0.6 | 0.8 | 0.2 | 0.2 | 0.1 | 0 |
| 5 June | 224 | 49 | 9.30E-05 | 2.00E-05 | 2.80E-05 | 6.00E-06 | 8.30E-06 | 1.80E-06 | 2.8 | 0.6 | 0.8 | 0.2 | 0.2 | 0.1 | 0 |
| 6 June | 226 | 49 | 9.30E-05 | 2.00E-05 | 2.80E-05 | 5.90E-06 | 8.40E-06 | 1.80E-06 | 2.8 | 0.6 | 0.8 | 0.2 | 0.3 | 0.1 | 0 |
| 7 June | 228 | 48 | 9.40E-05 | 2.00E-05 | 2.80E-05 | 5.90E-06 | 8.50E-06 | 1.80E-06 | 2.8 | 0.6 | 0.8 | 0.2 | 0.3 | 0.1 | 0 |
| 8 June | 230 | 48 | 9.50E-05 | 1.90E-05 | 2.80E-05 | 5.80E-06 | 8.50E-06 | 1.70E-06 | 2.8 | 0.6 | 0.9 | 0.2 | 0.3 | 0.1 | 0 |
| 9 June | 231 | 47 | 9.50E-05 | 1.90E-05 | 2.90E-05 | 5.80E-06 | 8.60E-06 | 1.70E-06 | 2.9 | 0.6 | 0.9 | 0.2 | 0.3 | 0.1 | 0 |
| 10 June | 233 | 47 | 9.60E-05 | 1.90E-05 | 2.90E-05 | 5.70E-06 | 8.60E-06 | 1.70E-06 | 2.9 | 0.6 | 0.9 | 0.2 | 0.3 | 0.1 | 0 |
| 11 June | 234 | 46 | 9.60E-05 | 1.90E-05 | 2.90E-05 | 5.60E-06 | 8.70E-06 | 1.70E-06 | 2.9 | 0.6 | 0.9 | 0.2 | 0.3 | 0.1 | 0 |
| 12 June | 236 | 46 | 9.70E-05 | 1.90E-05 | 2.90E-05 | 5.60E-06 | 8.70E-06 | 1.70E-06 | 2.9 | 0.6 | 0.9 | 0.2 | 0.3 | 0.1 | 0 |
| 13 June | 237 | 46 | 9.70E-05 | 1.80E-05 | 2.90E-05 | 5.50E-06 | 8.80E-06 | 1.70E-06 | 2.9 | 0.6 | 0.9 | 0.2 | 0.3 | 0.0 | 0 |
| 14 June | 238 | 45 | 9.80E-05 | 1.80E-05 | 2.90E-05 | 5.50E-06 | 8.80E-06 | 1.60E-06 | 2.9 | 0.5 | 0.9 | 0.2 | 0.3 | 0.0 | 0 |
| 15 June | 239 | 45 | 9.80E-05 | 1.80E-05 | 2.90E-05 | 5.40E-06 | 8.80E-06 | 1.60E-06 | 2.9 | 0.5 | 0.9 | 0.2 | 0.3 | 0.0 | 0 |
| 16 June | 240 | 44 | 9.80E-05 | 1.80E-05 | 3.00E-05 | 5.40E-06 | 8.90E-06 | 1.60E-06 | 3.0 | 0.5 | 0.9 | 0.2 | 0.3 | 0.0 | 0 |
| 17 June | 241 | 44 | 9.90E-05 | 1.80E-05 | 3.00E-05 | 5.30E-06 | 8.90E-06 | 1.60E-06 | 3.0 | 0.5 | 0.9 | 0.2 | 0.3 | 0.0 | 0 |
| 18 June | 241 | 43 | 9.90E-05 | 1.80E-05 | 3.00E-05 | 5.30E-06 | 8.90E-06 | 1.60E-06 | 3.0 | 0.5 | 0.9 | 0.2 | 0.3 | 0.0 | 0 |
| 19 June | 242 | 43 | 9.90E-05 | 1.70E-05 | 3.00E-05 | 5.20E-06 | 8.90E-06 | 1.60E-06 | 3.0 | 0.5 | 0.9 | 0.2 | 0.3 | 0.0 | 0 |
| 20 June | 242 | 42 | 9.90E-05 | 1.70E-05 | 3.00E-05 | 5.20E-06 | 8.90E-06 | 1.50E-06 | 3.0 | 0.5 | 0.9 | 0.2 | 0.3 | 0.0 | 0 |
| 21 June | 243 | 42 | 1.00E-04 | 1.70E-05 | 3.00E-05 | 5.10E-06 | 9.00E-06 | 1.50E-06 | 3.0 | 0.5 | 0.9 | 0.2 | 0.3 | 0.0 | 2 |
| 22 June | 243 | 42 | 1.00E-04 | 1.70E-05 | 3.00E-05 | 5.10E-06 | 9.00E-06 | 1.50E-06 | 3.0 | 0.5 | 0.9 | 0.2 | 0.3 | 0.0 | 2 |
| 23 June | 244 | 41 | 1.00E-04 | 1.70E-05 | 3.00E-05 | 5.00E-06 | 9.00E-06 | 1.50E-06 | 3.0 | 0.5 | 0.9 | 0.2 | 0.3 | 0.0 | 2 |
| 24 June | 244 | 41 | 1.00E-04 | 1.70E-05 | 3.00E-05 | 5.00E-06 | 9.00E-06 | 1.50E-06 | 3.0 | 0.5 | 0.9 | 0.1 | 0.3 | 0.0 | 2 |
| 25 June | 244 | 40 | 1.00E-04 | 1.60E-05 | 3.00E-05 | 4.90E-06 | 9.00E-06 | 1.50E-06 | 3.0 | 0.5 | 0.9 | 0.1 | 0.3 | 0.0 | 0 |
| 26 June | 244 | 40 | 1.00E-04 | 1.60E-05 | 3.00E-05 | 4.90E-06 | 9.00E-06 | 1.50E-06 | 3.0 | 0.5 | 0.9 | 0.1 | 0.3 | 0.0 | 2 |
| 27 June | 244 | 40 | 1.00E-04 | 1.60E-05 | 3.00E-05 | 4.80E-06 | 9.00E-06 | 1.40E-06 | 3.0 | 0.5 | 0.9 | 0.1 | 0.3 | 0.0 | 2 |
| 28 June | 244 | 39 | 1.00E-04 | 1.60E-05 | 3.00E-05 | 4.80E-06 | 9.00E-06 | 1.40E-06 | 3.0 | 0.5 | 0.9 | 0.1 | 0.3 | 0.0 | 2 |
| 29 June | 243 | 39 | 9.90E-05 | 1.60E-05 | 3.00E-05 | 4.70E-06 | 8.90E-06 | 1.40E-06 | 3.0 | 0.5 | 0.9 | 0.1 | 0.3 | 0.0 | 2 |
| 30 June | 243 | 38 | 9.90E-05 | 1.60E-05 | 3.00E-05 | 4.70E-06 | 8.90E-06 | 1.40E-06 | 3.0 | 0.5 | 0.9 | 0.1 | 0.3 | 0.0 | 0 |
| 1 July | 243 | 38 | 9.90E-05 | 1.50E-05 | 3.00E-05 | 4.60E-06 | 8.90E-06 | 1.40E-06 | 3.0 | 0.5 | 0.9 | 0.1 | 0.3 | 0.0 | 0 |
| 2 July | 242 | 38 | 9.90E-05 | 1.50E-05 | 3.00E-05 | 4.60E-06 | 8.90E-06 | 1.40E-06 | 3.0 | 0.5 | 0.9 | 0.1 | 0.3 | 0.0 | 2 |
| 3 July | 242 | 37 | 9.90E-05 | 1.50E-05 | 3.00E-05 | 4.50E-06 | 8.90E-06 | 1.40E-06 | 3.0 | 0.5 | 0.9 | 0.1 | 0.3 | 0.0 | 2 |
| 4 July | 241 | 37 | 9.80E-05 | 1.50E-05 | 3.00E-05 | 4.50E-06 | 8.90E-06 | 1.30E-06 | 3.0 | 0.4 | 0.9 | 0.1 | 0.3 | 0.0 | 2 |
| 5 July | 241 | 37 | 9.80E-05 | 1.50E-05 | 2.90E-05 | 4.40E-06 | 8.80E-06 | 1.30E-06 | 2.9 | 0.4 | 0.9 | 0.1 | 0.3 | 0.0 | 2 |
| 6 July | 240 | 36 | 9.80E-05 | 1.50E-05 | 2.90E-05 | 4.40E-06 | 8.80E-06 | 1.30E-06 | 2.9 | 0.4 | 0.9 | 0.1 | 0.3 | 0.0 | 0 |
| 7 July | 239 | 36 | 9.80E-05 | 1.50E-05 | 2.90E-05 | 4.40E-06 | 8.80E-06 | 1.30E-06 | 2.9 | 0.4 | 0.9 | 0.1 | 0.3 | 0.0 | 2 |
| 8 July | 238 | 36 | 9.70E-05 | 1.40E-05 | 2.90E-05 | 4.30E-06 | 8.70E-06 | 1.30E-06 | 2.9 | 0.4 | 0.9 | 0.1 | 0.3 | 0.0 | 2 |
| 9 July | 238 | 35 | 9.70E-05 | 1.40E-05 | 2.90E-05 | 4.30E-06 | 8.70E-06 | 1.30E-06 | 2.9 | 0.4 | 0.9 | 0.1 | 0.3 | 0.0 | 2 |
| 10 July | 237 | 35 | 9.60E-05 | 1.40E-05 | 2.90E-05 | 4.20E-06 | 8.70E-06 | 1.30E-06 | 2.9 | 0.4 | 0.9 | 0.1 | 0.3 | 0.0 | 2 |
| 11 July | 236 | 35 | 9.60E-05 | 1.40E-05 | 2.90E-05 | 4.20E-06 | 8.60E-06 | 1.30E-06 | 2.9 | 0.4 | 0.9 | 0.1 | 0.3 | 0.0 | 0 |
| 12 July | 235 | 34 | 9.60E-05 | 1.40E-05 | 2.90E-05 | 4.20E-06 | 8.60E-06 | 1.20E-06 | 2.9 | 0.4 | 0.9 | 0.1 | 0.3 | 0.0 | 2 |
| 13 July | 234 | 34 | 9.50E-05 | 1.40E-05 | 2.90E-05 | 4.10E-06 | 8.60E-06 | 1.20E-06 | 2.9 | 0.4 | 0.9 | 0.1 | 0.3 | 0.0 | 2 |
| 14 July | 233 | 34 | 9.50E-05 | 1.40E-05 | 2.80E-05 | 4.10E-06 | 8.50E-06 | 1.20E-06 | 2.8 | 0.4 | 0.9 | 0.1 | 0.3 | 0.0 | 2 |
| 15 July | 231 | 33 | 9.40E-05 | 1.30E-05 | 2.80E-05 | 4.00E-06 | 8.50E-06 | 1.20E-06 | 2.8 | 0.4 | 0.8 | 0.1 | 0.3 | 0.0 | 2 |
| 16 July | 230 | 33 | 9.40E-05 | 1.30E-05 | 2.80E-05 | 4.00E-06 | 8.40E-06 | 1.20E-06 | 2.8 | 0.4 | 0.8 | 0.1 | 0.3 | 0.0 | 0 |
| 17 July | 229 | 33 | 9.30E-05 | 1.30E-05 | 2.80E-05 | 4.00E-06 | 8.40E-06 | 1.20E-06 | 2.8 | 0.4 | 0.8 | 0.1 | 0.3 | 0.0 | 2 |
| 18 July | 228 | 32 | 9.30E-05 | 1.30E-05 | 2.80E-05 | 3.90E-06 | 8.30E-06 | 1.20E-06 | 2.8 | 0.4 | 0.8 | 0.1 | 0.3 | 0.0 | 2 |
| 19 July | 227 | 32 | 9.20E-05 | 1.30E-05 | 2.80E-05 | 3.90E-06 | 8.30E-06 | 1.20E-06 | 2.8 | 0.4 | 0.8 | 0.1 | 0.2 | 0.0 | 2 |
| 20 July | 225 | 32 | 9.20E-05 | 1.30E-05 | 2.70E-05 | 3.80E-06 | 8.20E-06 | 1.20E-06 | 2.7 | 0.4 | 0.8 | 0.1 | 0.2 | 0.0 | 2 |
| 21 July | 224 | 31 | 9.10E-05 | 1.30E-05 | 2.70E-05 | 3.80E-06 | 8.20E-06 | 1.10E-06 | 2.7 | 0.4 | 0.8 | 0.1 | 0.2 | 0.0 | 0 |
| 22 July | 223 | 31 | 9.10E-05 | 1.30E-05 | 2.70E-05 | 3.80E-06 | 8.10E-06 | 1.10E-06 | 2.7 | 0.4 | 0.8 | 0.1 | 0.2 | 0.0 | 0 |
| 23 July | 221 | 31 | 9.00E-05 | 1.20E-05 | 2.70E-05 | 3.70E-06 | 8.10E-06 | 1.10E-06 | 2.7 | 0.4 | 0.8 | 0.1 | 0.2 | 0.0 | 2 |
| 24 July | 220 | 30 | 8.90E-05 | 1.20E-05 | 2.70E-05 | 3.70E-06 | 8.00E-06 | 1.10E-06 | 2.7 | 0.4 | 0.8 | 0.1 | 0.2 | 0.0 | 2 |
| 25 July | 218 | 30 | 8.90E-05 | 1.20E-05 | 2.70E-05 | 3.70E-06 | 8.00E-06 | 1.10E-06 | 2.7 | 0.4 | 0.8 | 0.1 | 0.2 | 0.0 | 2 |
| 26 July | 217 | 30 | 8.80E-05 | 1.20E-05 | 2.60E-05 | 3.60E-06 | 7.90E-06 | 1.10E-06 | 2.6 | 0.4 | 0.8 | 0.1 | 0.2 | 0.0 | 2 |
| 27 July | 215 | 30 | 8.80E-05 | 1.20E-05 | 2.60E-05 | 3.60E-06 | 7.90E-06 | 1.10E-06 | 2.6 | 0.4 | 0.8 | 0.1 | 0.2 | 0.0 | 0 |
| 28 July | 214 | 29 | 8.70E-05 | 1.20E-05 | 2.60E-05 | 3.60E-06 | 7.80E-06 | 1.10E-06 | 2.6 | 0.4 | 0.8 | 0.1 | 0.2 | 0.0 | 2 |
| 29 July | 212 | 29 | 1.60E-04 | 1.20E-05 | 4.80E-05 | 3.50E-06 | 1.40E-05 | 1.10E-06 | 4.8 | 0.4 | 1.4 | 0.1 | 0.4 | 0.0 | 2 |
| 30 July | 388 | 29 | 2.30E-04 | 1.20E-05 | 6.90E-05 | 3.50E-06 | 2.10E-05 | 1.00E-06 | 6.9 | 0.3 | 2.1 | 0.1 | 0.6 | 0.0 | 2 |
| 31 July | 563 | 28 | 2.90E-04 | 1.10E-05 | 8.60E-05 | 3.40E-06 | 2.60E-05 | 1.00E-06 | 8.6 | 0.3 | 2.6 | 0.1 | 0.8 | 0.0 | 2 |
| 1 August | 703 | 28 | 3.30E-04 | 1.10E-05 | 1.00E-04 | 3.40E-06 | 3.00E-05 | 1.00E-06 | 10.0 | 0.3 | 3.0 | 0.1 | 0.9 | 0.0 | 0 |
| 2 August | 812 | 28 | 3.70E-04 | 1.10E-05 | 1.10E-04 | 3.40E-06 | 3.30E-05 | 1.00E-06 | 11.0 | 0.3 | 3.3 | 0.1 | 1.0 | 0.0 | 2 |
| 3 August | 895 | 28 | 3.90E-04 | 1.10E-05 | 1.20E-04 | 3.30E-06 | 3.50E-05 | 1.00E-06 | 11.8 | 0.3 | 3.5 | 0.1 | 1.1 | 0.0 | 2 |
| 4 August | 954 | 27 | 4.10E-04 | 1.10E-05 | 1.20E-04 | 3.30E-06 | 3.70E-05 | 1.00E-06 | 12.2 | 0.3 | 3.7 | 0.1 | 1.1 | 0.0 | 2 |
| 5 August | 992 | 27 | 4.20E-04 | 1.10E-05 | 1.20E-04 | 3.30E-06 | 3.70E-05 | 9.90E-07 | 12.5 | 0.3 | 3.7 | 0.1 | 1.1 | 0.0 | 2 |
| 6 August | 1013 | 27 | 4.20E-04 | 1.10E-05 | 1.30E-04 | 3.30E-06 | 3.80E-05 | 9.80E-07 | 12.5 | 0.3 | 3.8 | 0.1 | 1.1 | 0.0 | 0 |
| 7 August | 1018 | 27 | 4.20E-04 | 1.10E-05 | 1.20E-04 | 3.20E-06 | 3.70E-05 | 9.70E-07 | 12.5 | 0.3 | 3.7 | 0.1 | 1.1 | 0.0 | 0 |
| 8 August | 1011 | 26 | 4.10E-04 | 1.10E-05 | 1.20E-04 | 3.20E-06 | 3.70E-05 | 9.60E-07 | 12.2 | 0.3 | 3.7 | 0.1 | 1.1 | 0.0 | 0 |
| 9 August | 994 | 26 | 4.00E-04 | 1.10E-05 | 1.20E-04 | 3.20E-06 | 3.60E-05 | 9.50E-07 | 11.9 | 0.3 | 3.6 | 0.1 | 1.1 | 0.0 | 0 |
| 10 August | 968 | 26 | 3.80E-04 | 1.00E-05 | 1.20E-04 | 3.10E-06 | 3.50E-05 | 9.40E-07 | 11.5 | 0.3 | 3.5 | 0.1 | 1.0 | 0.0 | 0 |
| 11 August | 937 | 26 | 3.70E-04 | 1.00E-05 | 1.10E-04 | 3.10E-06 | 3.30E-05 | 9.30E-07 | 11.1 | 0.3 | 3.3 | 0.1 | 1.0 | 0.0 | 0 |
| 12 August | 900 | 25 | 3.50E-04 | 1.00E-05 | 1.10E-04 | 3.10E-06 | 3.20E-05 | 9.20E-07 | 10.6 | 0.3 | 3.2 | 0.1 | 1.0 | 0.0 | 0 |
| 13 August | 860 | 25 | 3.40E-04 | 1.00E-05 | 1.00E-04 | 3.00E-06 | 3.00E-05 | 9.10E-07 | 10.1 | 0.3 | 3.0 | 0.1 | 0.9 | 0.0 | 0 |
| 14 August | 818 | 25 | 3.20E-04 | 1.00E-05 | 9.50E-05 | 3.00E-06 | 2.90E-05 | 9.00E-07 | 9.5 | 0.3 | 2.9 | 0.1 | 0.9 | 0.0 | 0 |
| 15 August | 775 | 25 | 3.00E-04 | 9.90E-06 | 9.00E-05 | 3.00E-06 | 2.70E-05 | 8.90E-07 | 9.0 | 0.3 | 2.7 | 0.1 | 0.8 | 0.0 | 0 |
| 16 August | 731 | 24 | 2.80E-04 | 9.80E-06 | 8.40E-05 | 2.90E-06 | 2.50E-05 | 8.80E-07 | 8.5 | 0.3 | 2.5 | 0.1 | 0.8 | 0.0 | 0 |
| 17 August | 688 | 24 | 2.60E-04 | 9.70E-06 | 7.90E-05 | 2.90E-06 | 2.40E-05 | 8.80E-07 | 7.9 | 0.3 | 2.4 | 0.1 | 0.7 | 0.0 | 0 |
| 18 August | 645 | 24 | 2.50E-04 | 9.60E-06 | 7.40E-05 | 2.90E-06 | 2.20E-05 | 8.70E-07 | 7.4 | 0.3 | 2.2 | 0.1 | 0.7 | 0.0 | 0 |
| 19 August | 603 | 24 | 2.30E-04 | 9.50E-06 | 6.90E-05 | 2.90E-06 | 2.10E-05 | 8.60E-07 | 6.9 | 0.3 | 2.1 | 0.1 | 0.6 | 0.0 | 0 |
| 20 August | 562 | 23 | 2.10E-04 | 9.50E-06 | 6.40E-05 | 2.80E-06 | 1.90E-05 | 8.50E-07 | 6.4 | 0.3 | 1.9 | 0.1 | 0.6 | 0.0 | 0 |
| 21 August | 523 | 23 | 2.00E-04 | 9.40E-06 | 6.00E-05 | 2.80E-06 | 1.80E-05 | 8.40E-07 | 6.0 | 0.3 | 1.8 | 0.1 | 0.5 | 0.0 | 0 |
| 22 August | 486 | 23 | 1.80E-04 | 9.30E-06 | 5.50E-05 | 2.80E-06 | 1.70E-05 | 8.30E-07 | 5.5 | 0.3 | 1.7 | 0.1 | 0.5 | 0.0 | 0 |
| 23 August | 451 | 23 | 1.70E-04 | 9.20E-06 | 5.10E-05 | 2.80E-06 | 1.50E-05 | 8.30E-07 | 5.1 | 0.3 | 1.5 | 0.1 | 0.5 | 0.0 | 0 |
| 24 August | 417 | 22 | 1.60E-04 | 9.10E-06 | 4.70E-05 | 2.70E-06 | 1.40E-05 | 8.20E-07 | 4.7 | 0.3 | 1.4 | 0.1 | 0.4 | 0.0 | 0 |
| 25 August | 386 | 22 | 1.50E-04 | 9.00E-06 | 4.40E-05 | 2.70E-06 | 1.30E-05 | 8.10E-07 | 4.4 | 0.3 | 1.3 | 0.1 | 0.4 | 0.0 | 0 |
| 26 August | 356 | 22 | 1.30E-04 | 8.90E-06 | 4.00E-05 | 2.70E-06 | 1.20E-05 | 8.00E-07 | 4.0 | 0.3 | 1.2 | 0.1 | 0.4 | 0.0 | 0 |
| 27 August | 328 | 22 | 1.20E-04 | 8.80E-06 | 3.70E-05 | 2.60E-06 | 1.10E-05 | 7.90E-07 | 3.7 | 0.3 | 1.1 | 0.1 | 0.3 | 0.0 | 0 |
| 28 August | 302 | 22 | 1.10E-04 | 8.70E-06 | 3.40E-05 | 2.60E-06 | 1.00E-05 | 7.90E-07 | 3.4 | 0.3 | 1.0 | 0.1 | 0.3 | 0.0 | 0 |
| 29 August | 278 | 21 | 1.00E-04 | 8.70E-06 | 3.10E-05 | 2.60E-06 | 9.40E-06 | 7.80E-07 | 3.1 | 0.3 | 0.9 | 0.1 | 0.3 | 0.0 | 0 |
| 30 August | 255 | 21 | 9.60E-05 | 8.60E-06 | 2.90E-05 | 2.60E-06 | 8.60E-06 | 7.70E-07 | 2.9 | 0.3 | 0.9 | 0.1 | 0.3 | 0.0 | 0 |
| 31 August | 234 | 21 | 8.80E-05 | 8.50E-06 | 2.60E-05 | 2.50E-06 | 7.90E-06 | 7.60E-07 | 2.6 | 0.3 | 0.8 | 0.1 | 0.2 | 0.0 | 0 |
| 1 September | 215 | 21 | 8.10E-05 | 8.40E-06 | 2.40E-05 | 2.50E-06 | 7.20E-06 | 7.60E-07 | 2.4 | 0.3 | 0.7 | 0.1 | 0.2 | 0.0 | 0 |
| 2 September | 197 | 21 | 7.40E-05 | 8.30E-06 | 2.20E-05 | 2.50E-06 | 6.60E-06 | 7.50E-07 | 2.2 | 0.2 | 0.7 | 0.1 | 0.2 | 0.0 | 0 |
| 3 September | 180 | 20 | 6.80E-05 | 8.20E-06 | 2.00E-05 | 2.50E-06 | 6.10E-06 | 7.40E-07 | 2.0 | 0.2 | 0.6 | 0.1 | 0.2 | 0.0 | 0 |
| 4 September | 165 | 20 | 6.20E-05 | 8.20E-06 | 1.90E-05 | 2.40E-06 | 5.60E-06 | 7.30E-07 | 1.9 | 0.2 | 0.6 | 0.1 | 0.2 | 0.0 | 0 |
| 5 September | 151 | 20 | 5.60E-05 | 8.10E-06 | 1.70E-05 | 2.40E-06 | 5.10E-06 | 7.30E-07 | 1.7 | 0.2 | 0.5 | 0.1 | 0.2 | 0.0 | 0 |
| 6 September | 138 | 20 | 5.10E-05 | 8.00E-06 | 1.50E-05 | 2.40E-06 | 4.60E-06 | 7.20E-07 | 1.5 | 0.2 | 0.5 | 0.1 | 0.1 | 0.0 | 0 |
| 7 September | 126 | 20 | 4.70E-05 | 7.90E-06 | 1.40E-05 | 2.40E-06 | 4.20E-06 | 7.10E-07 | 1.4 | 0.2 | 0.4 | 0.1 | 0.1 | 0.0 | 0 |
| 8 September | 115 | 19 | 4.30E-05 | 7.90E-06 | 1.30E-05 | 2.40E-06 | 3.90E-06 | 7.10E-07 | 1.3 | 0.2 | 0.4 | 0.1 | 0.1 | 0.0 | 0 |
| 9 September | 105 | 19 | 3.90E-05 | 7.80E-06 | 1.20E-05 | 2.30E-06 | 3.50E-06 | 7.00E-07 | 1.2 | 0.2 | 0.4 | 0.1 | 0.1 | 0.0 | 0 |
| 10 September | 96 | 19 | 3.60E-05 | 7.70E-06 | 1.10E-05 | 2.30E-06 | 3.20E-06 | 6.90E-07 | 1.1 | 0.2 | 0.3 | 0.1 | 0.1 | 0.0 | 0 |

S1, Scenario 1, without any preventive strategy; S2, Scenario 2, with vaccination against COVID-19; S3, Scenario 3, with a negative nucleic acid testing result within seven days before admission; S4, Scenario 4, with combined vaccination and nucleic acid testing; S5, Scenario 5, with combined nucleic acid testing and face mask wearing; S6, Scenario 6, with combined vaccination, nucleic acid testing, and face mask wearing.
